# Nitric Oxide as a Downstream Signaling Molecule in Brassinosteroid-Mediated Virus Susceptibility to Maize Chlorotic Mottle Virus in Maize

**DOI:** 10.3390/v11040368

**Published:** 2019-04-22

**Authors:** Ning Cao, Binhui Zhan, Xueping Zhou

**Affiliations:** 1 State Key Laboratory for Biology of Plant Disease and Insect Pest, Institute of Plant Protection, Chinese Academy of Agricultural Sciences, Beijing 100193, China; cang327@126.com; 2 State Key Laboratory for Rice Biology, Institute of Biotechnology, Zhejiang University, Hangzhou 310058, China

**Keywords:** maize chlorotic mottle virus, brassinosteroid, nitric oxide, virus susceptibility

## Abstract

Maize chlorotic mottle virus (MCMV) infection causes growth abnormalities in maize. Transcriptome sequencing was conducted to compare the global gene expression of MCMV-inoculated plants with that of mock-inoculated plants. Data analyses showed that brassinosteroid (BR)-associated genes were upregulated after MCMV infection. Exogenous 2,4-epibrassinolide (BL) or brassinazole (BRZ) applications indicated that BR pathway was involved in the susceptibility to MCMV infection. In addition, treatment of BL on maize induced the accumulation of nitric oxide (NO), and the changes of NO content played positive roles in the disease incidence of MCMV. Moreover, MCMV infection was delayed when the BL-treated plants were applied with NO scavenger, which suggested that BR induced the susceptibility of maize to MCMV infection in a NO-dependent manner. Further investigation showed the maize plants with knock-down of *DWARF4* (*ZmDWF4*, a key gene of BR synthesis) and nitrate reductase (*ZmNR*, a key gene of NO synthesis) by virus-induced gene silencing displayed higher resistance to MCMV than control plants. Taken together, our results suggest that BR pathway promotes the susceptibility of maize to MCMV in a NO-dependent manner.

## 1. Introduction

Plants, most of which exhibit sessile lifestyles, encounter numerous biotic and abiotic stresses, including pathogenic challenges [1]. Virus is one of the most important pathogens infecting plants, which causes massive losses of crops yield. Virus invasions change host gene expression patterns, reprogram plant-signaling controls, disrupt central cellular metabolic pathways, and effectively evade defense responses leading to host susceptibility [2]. When the homeostasis of plants is broken by viral invasion, plants can adjust defense strategies to suppress virulence according to the type of pathogens. In fact, decades of studies have proved that plant hormones are closely associated with the process of plant innate antiviral immune responses [3,4]. With a large diversity in virus form, replication, and pathogenic effect, it is likely that different viruses in different hosts have varied effects on plant hormone metabolism [5]. Viral infections result in hormonal disruption because of the simultaneous induction of several antagonistic hormones in susceptible plants, while these antagonistic hormones may exhibit some sequential accumulation in resistant lines [6]. Moreover, extreme severity of symptoms caused by many viruses could cause changes in hormone concentration that have no involvement the in control of the host-virus interaction, which give great prominence to the roles of hormones in plant-virus interactions [5].

Brassinosteroids (BRs), a class of steroid phytohormones, play essential roles in variety of plant growth and development processes [7]. The bioactive BRs signal through receptors and co-receptors to modulate specific transcription factors, which furthermore regulate thousands of target genes [8,9]. Global gene expression studies demonstrated that BR can regulate up to 4000–5000 genes at different developmental stages, which proved the complexity of BR transcriptional network and the versatility of its biological functions [10]. In addition to their roles in plant growth and development, BRs are also involved in response to biotic and abiotic stresses. Notably, several recent studies revealed BRs participated in pathogen infection. In some cases, BRs have the positive effects on plant immunity, such as exogenous application of BRs on plants induced the resistance of *Arabidopsis* to cucumber mosaic virus (CMV) [11,12], oilseed rape to *Pseudomonas syringae* [13], and tobacco to tobacco mosaic virus (TMV) [14,15], etc. On the other cases, BRs render the susceptibility of plants to pathogen infection. For example, the treatment of BRs on rice enhance its susceptibility to rice black-streaked dwarf virus (RBSDV) [3], brown planthopper [16], and root-knot nematodes [17], etc. However, very limited information on the roles of BRs in plant immunity, which need more investigations.

Free radical Reactive nitrogen species (RNS), particularly nitric oxide (NO), are a family of antimicrobial molecules derived from nitric oxide free radical (NO·) and superoxide (O_2_^−^), which have been proved to be involved in the acclimation to different stresses including pathogen attacks [14,18]. NO is a small, potentially toxic, relatively unstable free radical gas with diatomic free radical [19]. Due to its highly lipophilic nature, NO often acts as a biological messenger as it can diffuse through cell membranes without the aids of specific membrane transporters [20]. During the plant-pathogen interactions, NO often makes contributions to the local and systemic induction of defense genes [21]. One hand, NO can be involved in direct defense mechanisms, such as cross-linking of plant cell walls, acting as antimicrobial agents, etc. On the other hand, NO acts as an active participant in signal transduction pathways, which can introduce specific post-translational modifications and lead to corresponding responses [22,23]. In terms of plant-virus interaction, some previous studies have shown that NO was a proper signaling molecule during virus infection, and fluorescent detection of NO made it possible to demonstrate its presence within virus-infecting plant tissues [12,23]. Moreover, recent researches indicated that *Arabidopsis* plants had the highest CMV replication and suffered more damages after NO scavenger or NO synthetic inhibitor treatment [12]. However, little is known about the roles of this gaseous free radical NO and its related molecules during virus infection in plants.

Maize chlorotic mottle virus (MCMV) is a single-stranded, positive-sense RNA virus belonging to the *Machlomovirus* genus of *Tombusviridae* [24]. The genome contains 4,437 nucleotides encoding 7 putative proteins including P32, P50, P111, P7a, P7b, P31, and coat protein (CP) [25]. During infection, MCMV can generate two major subgenomic RNAs of 1.47 and 0.34 kb in length [26]. MCMV is mainly transmitted to maize, sugarcane and other moncotyledon plants by mechanical inoculation, seeds, and insect vectors [27,28,29,30]. Single infections of MCMV on maize cause only mild symptoms [31], while co-infections with the viruses in the family *Potyviridae*, such as sugarcane mosaic virus, maize dwarf mosaic virus, or wheat streak mosaic virus, would cause serious disease, maize lethal necrosis disease (MLND), resulting in heavy yield losses of maize [32]. MCMV infection causes growth abnormalities in plants, but the mechanisms of these abnormalities during MCMV infection have not been well investigated.

To better understand the mechanisms of maize-MCMV interactions, we used a high-throughput sequencing approach to compare the global gene expression of MCMV-inoculated maize plants with that of mock-inoculated plants. We found that BR pathway was significantly altered after MCMV infection and exogenous BR applications showed higher susceptibility of maize to MCMV. In contrast, the plants with knock-down of *DWARF4* (*ZmDWF4*, a key gene of BR synthesis) displayed higher resistance to MCMV than control plants. Besides, NO was also required for efficient MCMV accumulation. Nitrate reductase (NR) is a key enzyme of NO synthesis in maize. Further investigation showed that the role of BR in promoting MCMV susceptibility was disappeared in *ZmNR*-silenced plants or in NO scavenger-treated plants. Taken together, our research demonstrated that BR promoted the susceptibility of maize to MCMV in a NO-dependent manner and provided insights into the complexity of crosstalk between plant hormones and RNS in virus-host interaction.

## 2. Materials and Methods

### 2.1. Plant Growth and Virus Inoculations

Maize (*Zea mays* L.) plants (cv. inbred line 2238/Va35) were grown inside growth chambers (24 °C day and 22 °C night, 16 h light and 8 h dark cycles). The MCMV isolate YN2 (MCMV-YN2) was propagated in maize plants, and the crude extracts from MCMV-infected leaves (at a ratio of 1:10 (w/v) in 0.01 M phosphate butter (PB, PH 7.0) were used to inoculate the first true leaves of 7-day-old maize seedlings, as previously described [33,34]. Maize seedlings of the same age were mock-inoculated with PB as control plants (CK). The first systematically infected leaves (SL1) were collected 4, 7, and 10 days post inoculation (dpi).

### 2.2. RNA-Seq Library Construction and Sequencing

The leaf samples from CK and MCMV-inoculated plants were ground immediately in liquid nitrogen and total RNAs were extracted by TRIzol reagent (Invitrogen, Carlsbad, CA, USA) following the manufacturer’s instructions. The quantity and purity of RNA, addition of adapters, size selection, and RNA-seq were performed by Novogene Bioinformatics Technology (Beijing, China). Sequencing libraries were constructed with NEBNext^®^ Ultra^TM^ RNA Library Prep Kit for Illumina^®^ (NEB, Ipswich, MA, USA) following manufacturer’s recommendations and index codes were added to attribute sequences to each sample. After cluster generation, the library preparations were sequenced on an Illumina Hiseq platform and 125 bp/150 bp single-end reads were generated. The raw reads were processed by adapter trimming, quality trimming, and length trimming to produce clean reads. The mapping of clean reads onto the maize B73 reference genome was conducted using Bowtie v2.2.3 software (https://sourceforge.net/projects/bowtie-bio/) with the default parameters [35].

To ensure true representation of fragment gene expression levels, read numbers and gene lengths were normalized using fragments per kilobase of transcript per million fragments mapped (FPKM). DESeq was used to calculate the differential expression of contigs and the *p*-value was obtained based on the method of negative binomial distribution [36]. The *p*-value was adjusted with a false-discovery rate (FDR) correction for multiple testing by the Benjamini-Hochberg method [37]. A difference in gene expression between mock-inoculated and MCMV-inoculated plants was considered significant when the absolute value of log_2_ (fold change) ratio was ≥1 and *q*-value ≤ 0.05 (corrected *p*-value) [38]. The statistical enrichments of differential expression genes were tested in Gene Ontology (GO) and Kyoto Encyclopedia of Genes and Genomes (KEGG) pathways using *p*-value ≤ 0.05 as cut-off [39,40].

### 2.3. Quantitative Analysis of BR Concentrations

The first systematically infected leaves of mock-inoculated and MCMV-inoculated plants were collected at 4, 7, and 10 dpi, ground in liquid nitrogen and then used for BRs extraction and analysis. The extraction and concentration analysis of BRs from maize seedlings were performed using Plant Brassinosteroids ELISA Kit (Jianglaibio, Shanghai, China) according to the manufacturer’s protocol. Three independent experiments were conducted each with at least four biological replicates.

### 2.4. Measurement of Endogenous NO

For fluorescence microscopy, NO was visualized using the NO fluorescent probe 4-Amino-5-methylamino-2’,7’-difluorofluorescein diacetate (DAF-FM-DA) (Nanjing KeyGen Biotec, Nanjing, China). Leaf fragments were pre-loaded with 5 μM DAF-FM-DA for 30 min in PB (pH 7.4) in darkness at 25 °C. Then, the leaves were washed three times at 25 °C using PB for 5 min each time, and then visualized using a Carl Zeiss laser scanning confocal microscopy 880 (LSMT-PMT, Carl Zeiss, Oberkochen, Germany) with the excitation at 488 nm and emission at 515 nm. To obtain the quantitative data, experiments were performed with the strictly identical confocal settings (e.g., laser power, gain factor, zoom, and emission wave length reception). Graphs represent quantifications from three independent biological experiments. The fluorescence intensity of the individual leaf was determined using Image J software (http://imagej.net/). At least three leaves from different plants were analyzed per experiment. In addition, endogenous NO production was also quantified using the Griess reagent according to the manufacturer’s instruction (Promega, Madison, WI, USA).

### 2.5. Hormone Treatments

2,4-Epibrassinolide (the most active brassinosteroid, BL) (Sigma-Aldrich, St Louis, MO, USA), brassinazole (the BR biosynthesis inhibitor, BRZ) (Sigma-Aldrich, St Louis, MO, USA), sodium nitroprusside (NO donor, SNP) (Sigma-Aldrich, St Louis, MO, USA) and 2-(4-carboxyphenyl)-4,4,5,5-tetramethyl-imidazoline-1-1-oxyl-3-oxide (NO scavenger, c-PTIO) (Sigma-Aldrich, St Louis, MO, USA) were dissolved in 100% ethanol as stock solutions and diluted with sterile distilled water containing 0.02% Tween-20. The 0.02% Tween with the same volume of ethanol was used as the mock control. To evaluate the effects of BL, BRZ, SNP and c-PTIO on MCMV infection, maize seedlings were sprayed with the corresponding solution 12 h before the mechanical inoculation of MCMV. The concentrations used are as follows: 500 μM BL, 1 μM BRZ, 200 μM SNP, and 200 μM c-PTIO. Three independent experiments were performed and at least 30 maize seedlings were conducted per experiment. The numbers of healthy and diseased plants in each experiment were recorded periodically.

### 2.6. Brome Mosaic Virus (BMV)-Based Virus-Induced Gene Silencing (VIGS) in Maize

The fragments representing partial sequences of *ZmDWF4* (MaizeGDB Zm00001d028325) (219 bp) and *ZmNR* (MaizeGDB Zm00001d049995) (236 bp) genes were PCR-amplified with cDNA using primers shown in Table S1. The fragments were digested with *Nco*I and *Avr*II restriction enzymes (NEB Inc., Beverly, MA, USA) and then cloned individually into the pC13/F3-13m vector [41]. The resulting constructs were named as pC13/F3-13m: *ZmDWF4* and pC13/F3-13m: *ZmNR*, respectively. These two constructs were sequenced before further use.

*Agrobacterium tumefaciens* cultures carrying pC13/F1 + 2, pC13/F3-13m: *ZmDWF4*, pC13/F3-13m: *ZmNR* or pC13/F3-13m: *GFP* were prepared and infiltrated into *Nicotiana benthamiana* leaves as described by Zhu et al. [34]. At 3 dpi, BMV virions were isolated from the agroinfiltrated *N. benthamiana* leaves and rub-inoculated into the maize seedlings (cv. Va 35), as previously described [34].

### 2.7. Quantitative Reverse Transcription Polymerase Chain Reaction (RT-qPCR)

About 2 μg of total RNA was treated with DNase I and then transcribed to cDNA using PrimeScript^TM^ RT reagent Kit with gDNA Eraser (TaKaRa, Dalian, Japan) according to the manufacturer’s protocol. As previously reported, the RT-qPCR was performed in triplicates using a Roche Light Cycler 96 system (Roche Applied Science, Basel, Switzerland) under the following program: 30 s at 95 °C, 45 cycles of 5 s at 95 °C, 30 s at 58 °C, and 10 s at 72 °C [33]. The specificity of the primer pairs was verified by the RT-qPCR dissociation curve and further confirmed by PCR visualized on a 1% agarose gel. The relative expression levels were calculated using the comparative Cq (2^-ΔΔCq^) method. Maize *ZmUBI* (ubiquitin) was used as an internal standard. The information of the primers used in the RT-qPCR experiments were listed in supplementary Table S1.

### 2.8. Western Blot Analysis

Total leaf proteins preparation and electrophoresis were performed as previously described [33]. The monoclonal antibody to MCMV CP was used to detect the accumulation of MCMV in the samples. The detection signal was visualized with a High-sig ECL Western Blotting Substrate (Tanon, Shanghai, China). The relative quantification of the proteins in each gel lane was calculated using the Image J image analysis tool, as previously described [42].

### 2.9. Statistical Analysis

Significant differences between treatments were determined by analysis of variance (ANOVA) with least significant difference (LSD) test at the level of *p*-value ≤ 0.05. All analyses were performed using Statistical Product and Service Solutions (SPSS) 16.0 (IBM, Florida, USA) (https://www.ibm.com/analytics/spss-statistics-software).

## 3. Results

### 3.1. The Phenotypes of MCMV-Infected Maize

**Figure 1:**
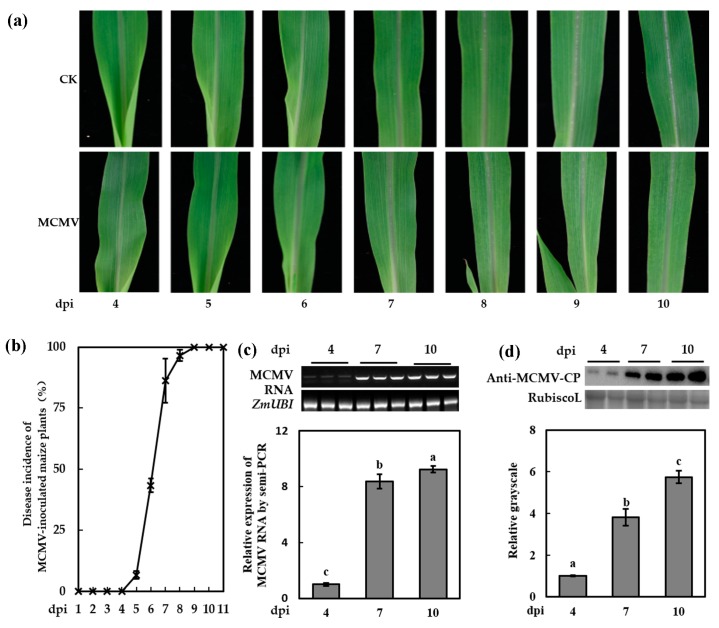
Symptom expression in the first systematically infected leaves (SL1) of maize chlorotic mottle virus (MCMV)-inoculated plants. (**a**) Phenotypes shown in the SL1 of MCMV-inoculated plants in a time course. (**b**) Disease incidence of MCMV-inoculated maize plants in a time course. (**c**) Semi-quantitative RT-PCR for the transcript level of MCMV RNA in the SL1 of MCMV-inoculated plants at 4, 7, and 10 days post inoculation (dpi). (**d**) Representative western blot analyses of MCMV accumulation in the SL1 of MCMV-inoculated plants at 4, 7, and 10 dpi. The relative grayscales were obtained through analyses of immunoblotting bands by ImageJ. Graphs represent quantifications of three independent experiments with at least 3 plants per experiment. Statistical analysis was performed using Statistical Product and Service Solutions (SPSS) 16.0 followed by analysis of variance (ANOVA) with least significant difference (LSD) test at the level of *p*-value ≤ 0.05. Significant difference between different samples was indicated in lowercase letters.

The first true leaves of 7-day-old maize seedlings (cv. 2238) were used for mechanically inoculation of MCMV. The symptoms development of mock-inoculated and MCMV-inoculated seedlings were periodically recorded, as shown in Figure 1a. The MCMV-inoculated plants showed light chlorotic mottle on the SL1 at 7 dpi (Figure 1a) and the disease incidence was over 94% (Figure 1b). By 9 dpi, all the MCMV-inoculated seedlings showed mottle mosaic symptoms. The semi-quantitative RT-PCR and western blot assays indicated that the accumulation of MCMV RNA and protein showed a similar tendency with the development of viral symptoms (Figure 1c,d). To elucidate the effect of MCMV infection on global transcript abundance in maize, we used high-throughput sequencing to compare the gene expression profiles of the SL1 of MCMV-inoculated plants with that of the mock-inoculated plants at 7 dpi, at which stage the mRNA and protein of MCMV were extensively accumulated and the symptoms were obvious.

### 3.2. Transcriptome Sequencing, Data Processing, and Differential Genes Expression Analyses

The SL1 of mock-inoculated and MCMV-inoculated plants were collected at 7 dpi for transcriptome sequencing in this study. To determine the accuracy of transcriptome data, 14 genes were selected to design specific primers and conduct RT-qPCR detection for validation. As illustrated in Figure S1 and Table S2, the RT-qPCR results were basically consistent with the transcriptome data, which confirmed the reliability of the transcriptome sequencing results.

**Figure 2:**
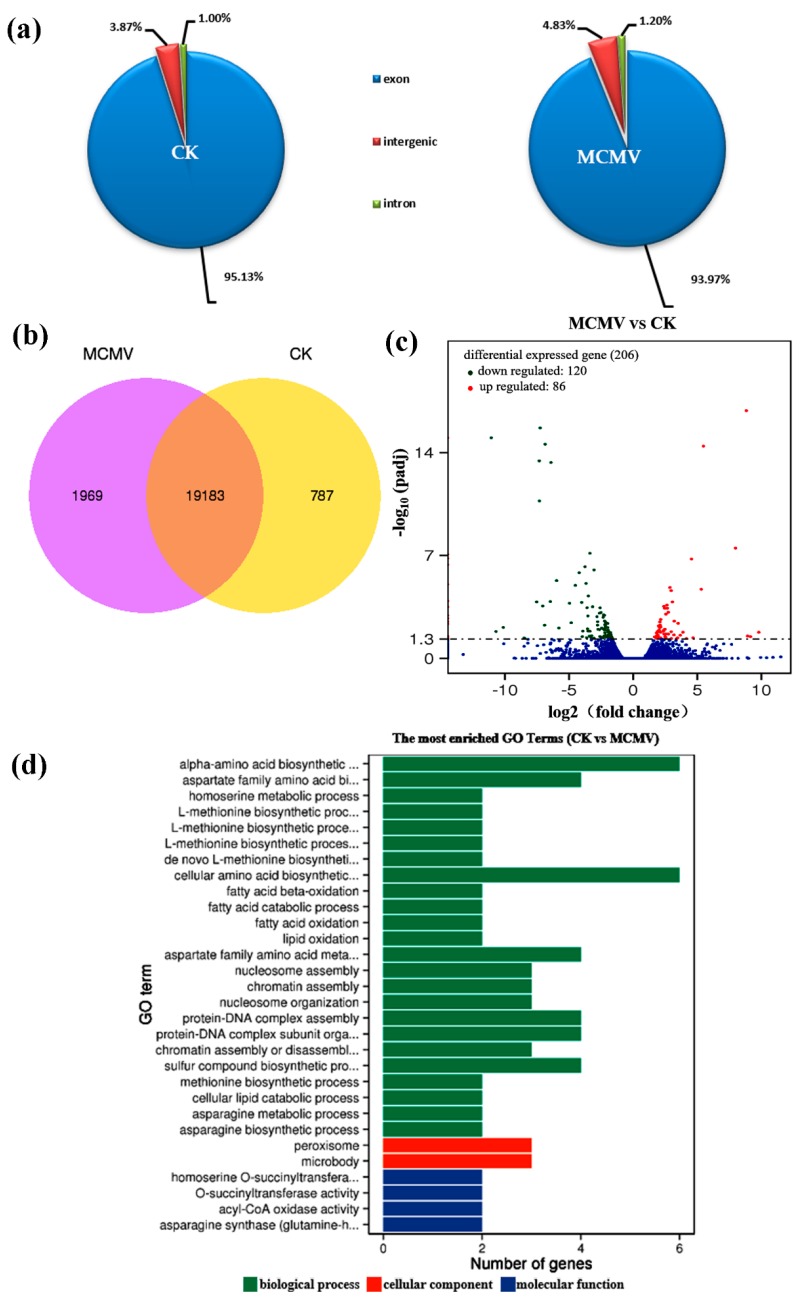
Overview of MCMV-induced responses in the maize transcriptome. (**a**) Graphical mapping summary with distribution of mapped reads across the regions of the reference genome. (**b**) Venn diagram illustrating the distribution of statistically significant changes in gene expression and their overlap among experimental groups. (**c**) Scatterplot analysis of differential gene expression in maize 7 dpi after MCMV infection compared with CK, a red dot stands for one up-regulated gene, a green dot for one down-regulated gene and a blue dot for one non-significantly changed gene. (**d**) Gene Ontology (GO) categories of enriched transcripts in maize leaves in response to MCMV infection determined using GO-seq. Data were taken from three biological replicates. CK: mock-inoculated maize; MCMV: MCMV-inoculated maize.

Subsequently, we used comparative transcriptome analysis of mock-inoculated and MCMV-inoculated maize leaves at 7 dpi to investigate maize-MCMV molecular interactions. We constructed 6 libraries prepared from 3 independent mock-inoculated maize plants (control group) and 3 independent MCMV-inoculated maize plants (experimental group). The raw sequence reads have been submitted to the NCBI Sequence Read Archive (SRA) (http://www.ncbi.nlm.nih.gov/sra) under accession number PRJNA527190 and SRP188502. After removing adaptors and reads of low-quality nucleotides, the clean reads for mock-inoculated and MCMV-inoculated samples were obtained, respectively (Table S3). The clean reads were mapped to the reference genome using HISAT [43]. The 84.93% of clean reads from mock-inoculated samples were mapped reads, and 80.75% of which were the uniquely mapped reads. The 83.27% of clean reads from MCMV-inoculated samples were mapped reads, and 80.14% of which were the uniquely mapped reads (Table S3). Next, GC contents of sequencing data from mock-inoculated and MCMV-inoculated plants were 59.15% and 57.93%, respectively. Moreover, the total mapped reads were compared to the maize genome, which showed that 95.13% of exons from mock-inoculated samples and 93.97% from MCMV-inoculated samples could be mapped to the genome assembly (Figure 2a). These results also showed that the sequence data produced from the libraries were suitable for data analyses.

To understand the transcriptome profiling of maize after MCMV infection, the assessment of the putative identities of the assembled unigenes was performed using reads per kilobase per million (RPKM) > 1 as a criterion for gene expression, based on sequence similarity search. With the clean reads obtained from mock-inoculated and MCMV-inoculated samples, a total of 21,939 genes were identified at 95% confidence levels (Figure 2b). In the distribution of the total identified genes, the proportion of distinct genes expressed only in mock-inoculated plants was 3.94% (1969/21,152), and in MCMV-inoculated plants was 9.31% (787/19,970). RPKM and differentially expressed genes (DEGs)-seq were used to calculate the abundance of gene expression and identify the significantly DEGs between mock-inoculated and MCMV-inoculated group [36]. With the restrictive conditions of absolute value of log_2_ (fold change) ≥ 1.0 and *q*-value ≤ 0.05, the expression of 206 genes were identified significantly changed (Figure 2c), of which 86 genes were upregulated and 120 genes were downregulated in MCMV-inoculated group compared with the mock-inoculated group. The DEGs were listed in Supplementary File 2.

To better understand the function of DEGs and their associated metabolism pathways, GO functional enrichment analysis and KEGG pathway enrichment analysis were conducted. The 132 annotated unigenes of 206 DEGs were assigned to 836 GO terms, and 107 of which were significantly enriched with *p*-value ≤ 0.05 (Supplementary File 3). All significantly enriched groups of GO terms were categorized into three types: 69 (64.5%) for biological processes, 11 (10.3%) for cellular components, and 27 (25.2%) for molecular functions. Of the 132 DEGs, 118 DEGs were associated with molecular function (GO: 0003674), 101 DEGs with biological process (GO: 0008150) and 55 DEGs with cellular component (GO: 0005575). The top 30 enriched GO terms were selected to show in Figure 2d. KEGG pathway analysis was also performed for DEGs to understand the interaction of genes and to further identify active biological pathways in response to MCMV infection in maize. In this assay, DEGs were assigned to 33 pathways and 8 pathways were significantly enriched with *p*-value ≤ 0.05 (Supplementary File 3), including fatty acid degradation, biosynthesis of unsaturated fatty acids, alpha-linolenic acid metabolism, photosynthesis-antenna proteins, fatty acid metabolism, nitrogen metabolism, and DNA replication peroxisome. Interestingly, we found that nitrogen metabolism pathway was significantly enriched during MCMV infection. The two genes encoding nitrite reductase (NiR) (maizeGDB Zm00001d018161) and carbonic anhydrase (maizeGDB Zm00001d005920) were down-regulated after MCMV infection in this pathway.

### 3.3. BR Induced the Susceptibility of Maize to MCMV Infection

**Figure 3:**
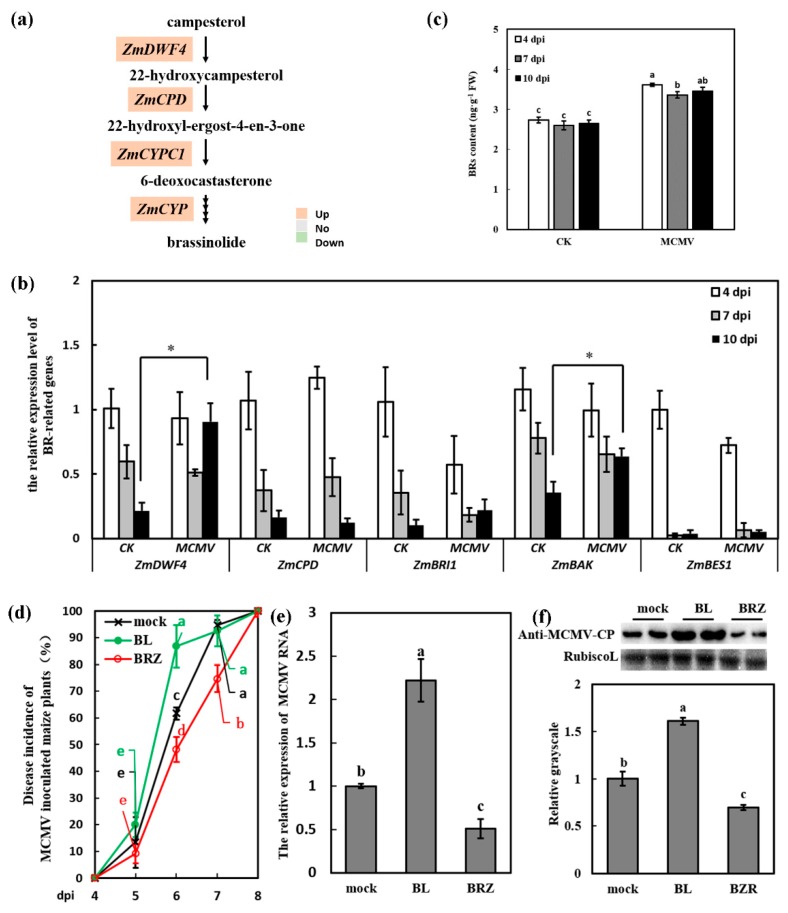
Brassinosteroid (BR) induced the susceptibility of maize to MCMV infection. (**a**) Transcriptional changes of BR biosynthesis pathway-associated genes during MCMV infection in maize. Highlighted in orange are up-regulated, while highlighted in green are down-regulated in the RNA-seq dataset. (**b**) RT-qPCR verification of the expression of BR pathway-related genes in response to MCMV infection at 4, 7, and 10 dpi in maize. (**c**) Endogenous BR concentration in the SL1 at 4, 7, 10 dpi in maize. (**d**) Disease incidence (%) of maize plants post MCMV inoculation with application of Tween-20 (0.02%, mock), 2,4-epibrassinolide (BL) (500 μM), and brassinazole (BRZ) (1 μM). Significant differences of disease incidence (%) between different treatments (mock, BL and BRZ) in the same dpi were indicated in lowercase letters (**e**) RT-qPCR analysis of the MCMV RNA accumulation after application of Tween-20 (0.02%, mock), BL (500 μM), and BRZ (1 μM). (**f**) Representative western blot showing MCMV accumulation with application of Tween-20 (0.02%, mock), BL (500 μM), and BRZ (1 μM). The grayscales of immunoblotting bands were determined by ImageJ. Graphs represent quantifications from three independent experiments with at least 3 plants per experiment. Statistical analysis was conducted using SPSS 16.0 followed by ANOVA with LSD test at the level of *p*-value ≤ 0.05. Significant difference between different samples was indicated in lowercase letters or *.

Plant hormones are not only essential for growth and development, but also play essential roles in plant defense responses [6], which have not been investigated in MCMV infection. The transcriptome data showed that the BR synthesis-related genes *ZmDWF4*, *constitutive photomorphogenesis and dwarfism* (*ZmCPD*, MaizeGDB Zm00001d028325), and two cytochrome P450 *ZmCYPC1* (MaizeGDB Zm00001d043317) and *ZmCYP* (MaizeGDB Zm00001d026083) were significantly increased after virus infection (Figure 3a). These results indicated that BR biosynthetic pathway might be regulated in response to MCMV infection. RT-qPCR was used to investigate the detailed transcript abundance of the BR pathways-related genes in response to MCMV infection. As shown in Figure 3b, most transcripts of the BR synthesis-related genes displayed a decreasing tendency along with the growth of both mock-inoculated and MCMV-inoculated plants. During the last period of MCMV infection (10 dpi), the transcript levels of *ZmDWF4* (which encoded the rate-limited enzyme in BR biosynthetic pathway) and *maize co-receptor BRI1-associated kinase* (*ZmBAK*) (BR signaling component) increased to 4.20 and 1.78 times in the SL1 of MCMV-inoculated plants compared with that of the mock-inoculated plants, respectively. Maize *Brassinosteroid Insensitive-1* (*ZmBRI1*) and *bri1 EMS SUPPRESSOR1* (*ZmBES*, BR receptors) showed no obvious changes after virus infection.

To confirm whether the transcript levels of these BR-related marker or BR biosynthesis genes in maize were correlated with actual hormone levels, the content of BRs was analyzed in the SL1 of MCMV-inoculated plants compared with mock-inoculated plants at 4, 7, and 10 dpi. The Figure 3c showed that the BRs levels of MCMV-inoculated plants were higher than that of mock-inoculated plants. For example, at 7 dpi, the BRs concentration was 2.60 ng·g^−1^ FW in the SL1 of mock-inoculated plants, and it was 3.36 ng·g^−1^ FW in the MCMV-inoculated plants, which showed a significant increase (Figure 3c). The increase of BRs levels after MCMV infection was basically consistent with upregulation of the BR synthesis-related genes in the transcriptome data and RT-qPCR results, which indicated that BR pathway was activated after virus infection and might play important roles during virus infection.

To get insights into the impact of BRs on MCMV infection, *Zea mays* (cv. 2238) plants were pretreated with 500 μM BL (the most active BR) or BRZ (BR biosynthesis inhibitor). Tween-20 (0.02%) was used as the hormone-free control (mock-treated plants). After 12 h treatment, the BL-treated and mock-treated maize seedlings were inoculated with MCMV. At least 30 seedlings were used for each treatment. The disease incidence of MCMV in plants was calculated periodically. As shown in Figure 3d, the infection of MCMV in BL-treated plants were obviously advanced compared with that of mock-treated and BRZ-treated plants. The disease incidence in BL-treated plants (86.79%) was significantly increased than that in mock-treated plants (61.67%) and the disease incidence in BRZ-treated plants (48.08%) was significantly decreased than that in mock-treated plants at 6 dpi. Moreover, the disease incidence in BL-treated plants (92.56%) was similar with that in mock-treated plants (94.67%) at 7dpi, while the disease incidence in BRZ-treated plants (74.73%) was still significantly lower than that in mock-treated and BL-treated plants. Virus accumulation was determined by RT-qPCR and Western blot analysis at 7 dpi. Our results showed that virus accumulation was significantly increased in the SL1 of BL-treated plants at RNA and protein levels (Figure 3e,f), compared with that of the mock-treated plants. Contrary to the results of BL treatment, the virus accumulation in the SL1 of BRZ-treated plants was significantly decreased compared with that of mock-treated plants at RNA and protein levels (Figure 3e,f). The results implied that BR pathway was associated with the susceptibility to MCMV infection.

### 3.4. BR Mediated the Susceptibility of Maize to MCMV Infection in the NO-Dependent Manner

**Figure 4:**
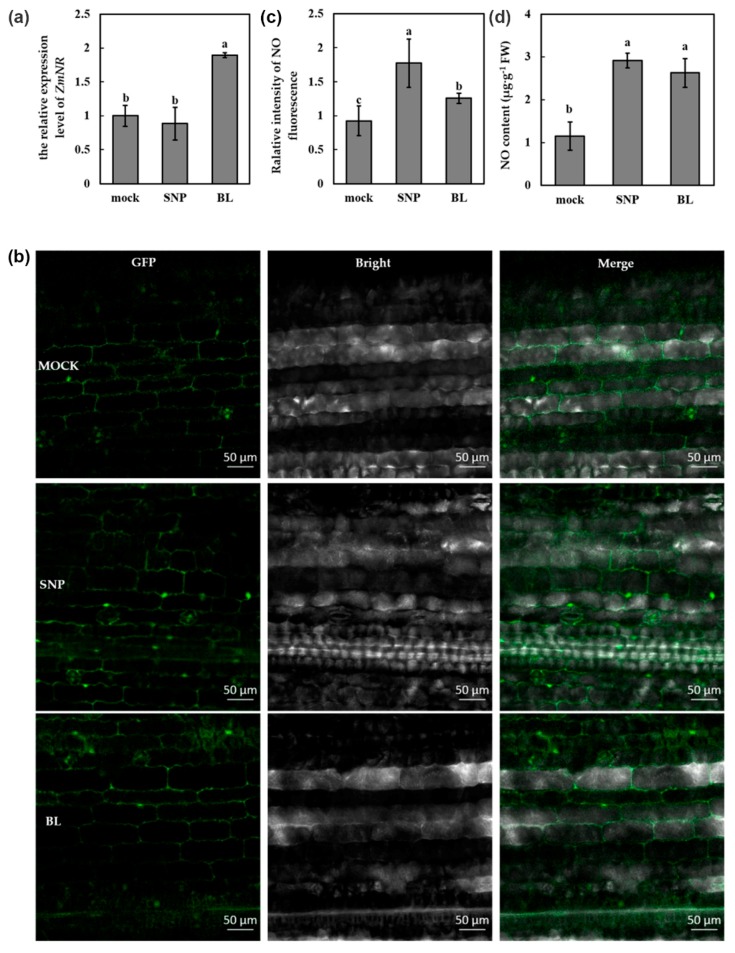
BR induced nitric oxide (NO) accumulation in maize. (**a**) RT-qPCR analysis of the transcript levels of NO synthetic gene *ZmNR*. (**b**) Image of NO level in leaves was detected by fluorescence resulting from DAF-FM-DA. Images were taken after 12 h of BL treatment using a fluorescence microscope. Scale bars = 50 μm. (**c**) Intensity of fluorescence of various treatments was analyzed with Image J software. (**d**) Quantitative measurements of NO content in the leaves. mock: leaves pretreated with Tween-20 (0.02%); SNP/BL: leaves pretreated with SNP, or BL. Data are means ± SD from three biological replicates. Statistical analysis was performed using SPSS 16.0 followed by ANOVA with LSD test at the level of *p*-value ≤ 0.05. Significant difference between different samples was indicated in lowercase letters.

Previous studies indicated that NO was a signaling molecule in BR-mediated virus resistance in dicots, such as tobacco and *Arabidopsis* [12,14]. To investigate the role of NO in BR-mediated susceptibility to MCMV, we detected the transcript abundance of NO synthetic gene *ZmNR* after BL treatment. The plants treated with 0.02% Tween-20 and SNP (NO donor) were used as mock and positive control, respectively. As shown in Figure 4a, the transcript of *ZmNR* was significantly induced after BL treatment, while there was no obvious change after SNP treatment. To further determine the contribution of BR to NO production, we detected the content of NO in maize leaves after BL treatment. NO were targeted with the fluorescent probe DAF-FM-DA and visualized by fluorescence microscopy. As shown in Figure 4b, the fluorescence intensity of BL-treated leaves was stronger than that of mock-treated leaves, and weaker than that of SNP-treated leaves. The observation and measurement were conducted with the same confocal settings. Analysis of the fluorescence intensity with Image J software demonstrated a significant increase after BL treatment (Figure 4c). At least three leaves from three different plants were analyzed per experiment. Another method with the Griess reagent was also applied to determine the NO content, which also indicated that the NO content was upregulated after BL treatment (Figure 4d). We also detected the transcript abundance of *ZmNR* and the content of NO in maize leaves after BRZ treatment. As shown in Figure S2, the transcript of *ZmNR* was significantly decreased and the NO content was downregulated after BRZ treatment, which was opposite to that of BL treatment. Together, these results demonstrated that BR could induce the increase of NO in maize.

**Figure 5:**
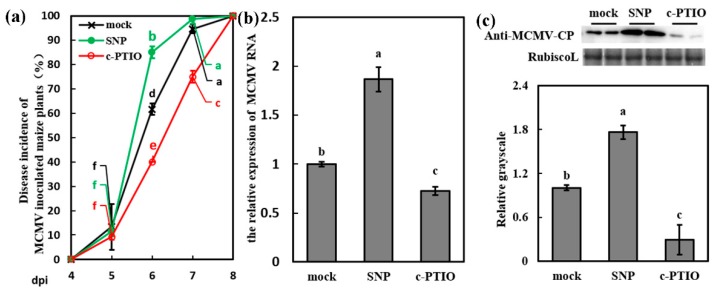
NO induced the susceptibility of maize to MCMV infection. (**a**) Disease incidence (%) of MCMV-inoculated maize plants after application of Tween-20 (0.02%, mock), SNP (200 μM), and c-PTIO (200 μM) with three independent replicates. Significant differences between different treatments (mock, SNP and c-PTIO) in the same dpi were indicated in lowercase letters. (**b**) RT-qPCR analysis of the MCMV RNA accumulation after application of Tween-20 (0.02%, mock), SNP (200 μM), and c-PTIO (200 μM). (**c**) Representative western blot showing MCMV accumulation after application of Tween-20 (0.02%, mock), SNP (200 μM), and c-PTIO (200 μM). The grayscales of immunoblotting bands were determined by ImageJ. Graphs represent the relative MCMV accumulation through quantifications of three independent experiments with at least three plants per experiment. Statistical analysis was performed using SPSS 16.0 followed by ANOVA with LSD test at the level of *p*-value ≤ 0.05. Significant difference between different samples was indicated in lowercase letters.

To get insights into the impact of NO on the maize defense response to MCMV, the maize seedlings were pretreated with SNP (NO donor) or c-PTIO (NO scavenger). After 12 h treatment, these SNP- and c-PTIO-treated plants were inoculated with MCMV. The effect of SNP/c-PTIO application has been confirmed by NO content determination (Figure 4a–d, Figure S3). There are significant differences of disease incidence (%) between SNP-treated (85.01%) and mock-treated plants (61.67%), between mock-treated and c-PTIO-treated plants (40.00%), as well as between SNP-treated and c-PTIO-treated plants at 6 dpi (Figure 5a). There was no significant difference between mock-treated and SNP-treated plants at 7 dpi, but the disease incidence in c-PTIO-treated plants (76.67%) was significantly lower than that in mock-treated (94.67%) and SNP-treated plants (98.81%) (Figure 5a). As shown in Figure 5b,c, the foliar application of SNP increased MCMV accumulation at 7 dpi, which showed a similar result as BL-treated plants. In the contrast, there was a significant decrease on virus accumulation in systematically infected leaves after c-PTIO treatment at the level of RNA and protein (Figure 5b,c).

**Figure 6:**
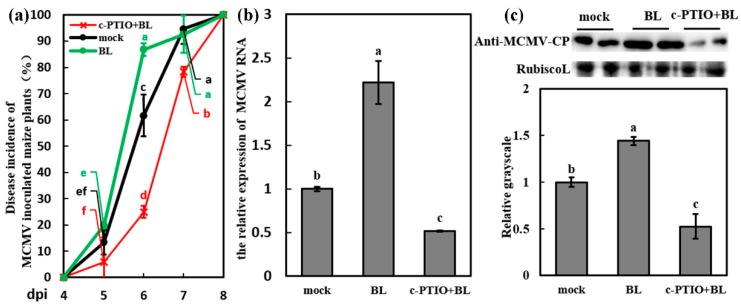
BR mediated the susceptibility to MCMV in a NO-dependent manner. (**a**) Disease incidence (%) of MCMV-inoculated maize plants after application of Tween-20 (0.02%, mock), BL (500 μM), and c-PTIO + BL (a mixture of 200 μM c-PTIO and 500 μM BL) with three independent replicates. Significant differences of disease incidence between different treatments (mock, BL and c-PTIO+BL) in the same dpi were indicated in lowercase letters. (**b**) RT-qPCR analysis of the MCMV RNA accumulation after application of Tween-20 (0.02%, mock), BL (500 μM), and c-PTIO + BL (200 μM c-PTIO and 500 μM BL). (**c**) Representative western blot showing MCMV accumulation after application of Tween-20 (0.02%, mock), BL (500 μM), and c-PTIO + BL (200 μM c-PTIO and 500 μM BL). The grayscales of immunoblotting bands were determined by ImageJ. Graphs represent the relative MCMV accumulation through quantifications of three independent experiments with at least three plants per experiment. Statistical analysis was performed using SPSS 16.0 followed by ANOVA with LSD test at the level of *p*-value ≤ 0.05. Significant difference between different samples was indicated in lowercase letters.

To further investigate whether NO was associated with BR-induced MCMV susceptibility, the c-PTIO was used as NO scavenger to decrease the NO content in BL-treated leaves. Figure S3 showed that the NO content was significantly reduced in simultaneously c-PTIO and BL-treated (c-PTIO + BL-treated) leaves, similar with the only c-PTIO-treated leaves, which revealed that BL treatment cannot rescue the decrease of NO content induced by c-PTIO. The c-PTIO + BL-treated seedlings was inoculated with MCMV and the disease incidence was counted, which showed that the infection of MCMV was delayed in c-PTIO + BL-treated plants compared with the BL-treated plants (Figure 6a). There are significant differences of disease incidence between c-PTIO + BL-treated and BL-treated plants at 6 dpi and 7 dpi. In addition, the disease incidence of c-PTIO + BL-treated plants was obviously decreased than mock-treated plants at 6 dpi and 7 dpi, which are similar to c-PTIO treatment. RT-qPCR and western blot assays also revealed that viral RNA and protein accumulation of c-PTIO + BL-treated plants were significantly decreased compared with BL-treated plants as well as mock-treated plants (Figure 6b,c), which were different with the upregulated viral accumulation after BL treatment compared with mock treatment. These results implied that NO was indispensable for BR-induced MCMV susceptibility.

### 3.5. ZmDWF4/ZmNR Silencing Inhibited MCMV Accumulation

**Figure 7:**
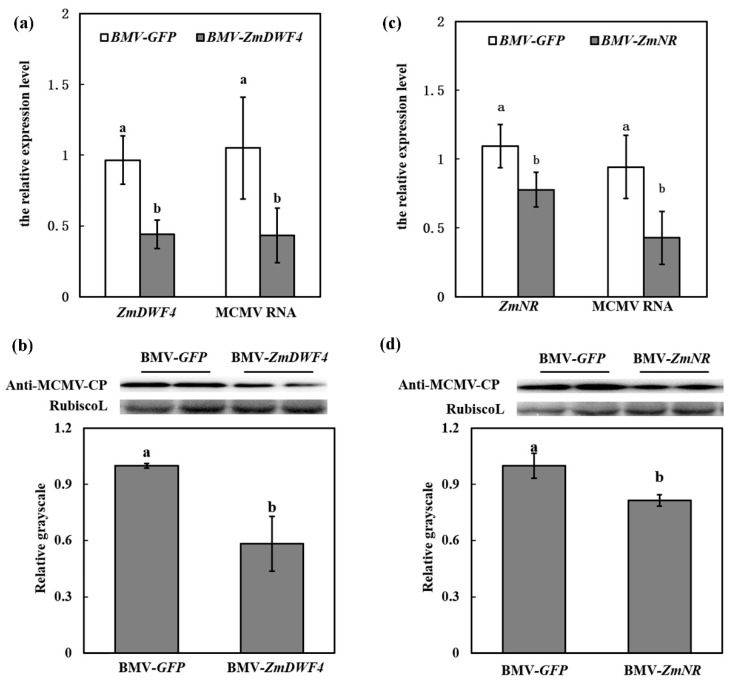
*ZmDWF4*/*ZmNR* silencing inhibited MCMV accumulation. (**a**) RT-qPCR analysis of the accumulation of *ZmDWF4* and MCMV RNA in the SL2 of BMV-GFP/MCMV and BMV-ZmDWF4/MCMV plants. (**b**) Representative Western blot indicated MCMV accumulation in the SL2 of BMV-*GFP*/MCMV and BMV-*ZmDWF4*/MCMV plants. The grayscales of immunoblotting bands from three independent experiments were determined by ImageJ. Graphs represent the relative MCMV accumulation through quantifications of three independent experiments with at least three plants per experiment. (**c**) RT-qPCR analysis of the accumulation of *ZmNR* and MCMV RNA in the SL2 of BMV-GFP/MCMV and BMV-*ZmNR*/MCMV plants. (**d**) Representative western blot indicated MCMV accumulation in the SL2 of BMV-*GFP*/MCMV and BMV-*ZmNR*/MCMV plants. The grayscales of immunoblotting bands from three independent experiments were determined by ImageJ. Graphs represent the relative MCMV accumulation through quantifications of three independent experiments with at least three plants per experiment. Statistical analysis was performed using SPSS 16.0 followed by ANOVA with LSD test at the level of *p*-value ≤ 0.05. Significant difference between different samples was indicated in lowercase letters.

To further investigate the involvement of BR pathway in the susceptibility of MCMV to maize, we used the BMV-VIGS system to knock down the expression of *ZmDWF4* in maize. To determine the accumulation of *ZmDWF4* in BMV-*ZmDWF4*-infected maize, the second systematically infected leaves (SL2) were harvested and subjected to RT-qPCR analysis at 7 dpi. Maize plants inoculated with BMV-*GFP* were used as controls. RT-qPCR results showed that the transcripts of *ZmDWF4* reduced 56% in BMV-*ZmDWF4*-infected plants, compared with the control BMV-*GFP*-infected plants (Figure 7a). At this time point, the BMV-inoculated plants were challenged with MCMV. At 7d after infection, the SL2 were collected to determine the RNA and protein accumulation of MCMV. Quantitative analyses revealed a 57% decrease in MCMV RNA and a 42% decrease in MCMV protein accumulation in *ZmDWF4*-silenced plants (BMV-*ZmDWF4*/MCMV) compared with the control plants (BMV-*GFP*/MCMV) (Figure 7a,b). The results confirmed that the BR pathway was essential to the susceptibility to MCMV infection.

We did the similar knock-down experiment with the *ZmNR* gene to further investigate the involvement of NO pathway in the susceptibility of maize to MCMV. RT-qPCR results showed that the relative transcript abundances of *ZmNR* reduced 22% in the SL2 in BMV-*ZmNR*-infected plants compared with the BMV*-GFP*-infected control plants (Figure 7c) at 7 dpi. In line with this, the content of NO in the SL2 of *ZmNR*-silenced plants was decreased (Figure S4). At this time point, the BMV-inoculated plants were challenged inoculated with MCMV. The accumulation of MCMV RNA in *ZmNR*-silenced plants (BMV-*ZmNR*/MCMV) were decreased by 57% at 7 dpi compared with the control plants (BMV-*GFP*/MCMV) (Figure 7c), which was accordant with the results of Western blot assay (Figure 7d). These results confirmed that NO pathway played an essential role in the susceptibility to MCMV infection.

Together, all these results demonstrated that the infection of MCMV induced the upregulation of BR pathway and BR increased the susceptibility of maize to MCMV. NO act as a crucial signaling molecule, which was essential for BR-induced systemic susceptibility to MCMV.

## 4. Discussion

In this study, we presented a detailed maize transcriptome analysis, focusing on DEGs in MCMV-inoculated and mock-inoculated leaves at 7 dpi. A total of 206 genes (86 induced and 120 repressed) were significantly changed in response to MCMV infection (Figure 2c). The trend towards downregulation of BR-related genes was alleviated in MCMV-inoculated plants, which further resulted in the accumulation of NO. The increased NO functioned as a downstream signal to enhance the susceptibility of maize to MCMV infection. Our results provide insights into the complexity of BR hormone and NO signaling molecule in virus-host interaction.

As plant hormones play crucial roles in defense and counter-defense of plant-virus interactions, identification of key hormones and their contributions during virus infection have been recognized as important aspects. Compared with the relatively deep understanding of positive roles of salicylic acid (SA) and jasmonic acid (JA) in response to virus infection, information regarding to BR is very limited. In the research of animal viruses, BR and their derivatives have antiviral activities against herpes simplex virus [44], arenaviruses [44], and measles virus [45] in cell cultures. Similar with the research on animal viruses, BRs in *Arabidopsis* and tobacco showed the enhanced resistance to CMV [11,12] and TMV [14,15]. BRI1, a leucine-rich repeat receptor-like kinase (LRR-RLK), is a BR receptor, which functions by interacting with the co-receptor BRI1-associated kinase 1 (BAK1) in a ligand-dependent manner [46]. BAK1 has been characterized as an essential factor for plant basal immunity during compatible interactions with RNA viruses. Compared with WT plants, the knock-out of *BAK1* in *Arabidopsis* results in higher accumulation of three different RNA viruses, turnip crinkle virus, TMV and oilseed rape mosaic virus [47]. However, contrary to the role of BR in virus resistance, several cases also report that BR mediate susceptibility to pathogen attack in rice, such as BR suppress rice defense against root-knot nematodes [17], BR mediated susceptibility to brown planthopper in rice [16], and BR enhanced susceptibility of rice to RBSDV infection [3]. In this study, we have suggested that BRs content were increased after MCMV infection in maize, the infection of MCMV was advanced by exogenous application of BL on maize plants and knock-down of *ZmDWF4* reduced MCMV accumulation. These results implied that BR played a negative role in the maize immunity to MCMV, which was similar with the several cases of pathogen-infected rice seedlings [3,16,17]. Together, the function of BR might play different roles in response to virus attack in different plants, which need more research.

In addition to hormones, plant responses to virus invasion are associated with accumulation of RNS [12]. NO is the representative component of RNS, which has been proved to play indispensable roles in signal transduction in response to biotic and abiotic stresses in plants [19]. During the last few years, the increasing researches have changed our views of RNS, from harmful byproducts to signaling molecules involved in many physiological processes in plants [19,48,49]. In addition, NO also participates in the plant immunity responses under pathogen attack. In terms of NO, BR was the most closely related hormone according to previous researches. With the assist of pharmacological and genetic methods, Deng et al. [14] found that BR can mediate systemic virus resistance in tobacco through a signaling pathway involved in H_2_O_2_ production and NO generation. Both NO and BR could mediate fungal endophyte-induced volatile oil production through protein phosphorylation pathways in *Atractylodes lancea* plantlets, and NO pathway might act as a downstream signaling event of BR pathway [50]. NO was involved in BR-induced alternative respiratory pathway capability which played essential roles in salt tolerance in *N. benthamiana* seedlings [51]. NO acts as a signaling molecule mediating BR-induced flavonoid biosynthesis in *Camellia sinensis* [52]. NO biosynthesis is involved in BR-mediated virus resistance to CMV in *Arabidopsis* [12]. Consistently, in our study, we also demonstrated that NO accumulation was induced by exogenous BR treatment in maize (Figure 4b–d), and NO accumulation was indispensable for BR-mediated MCMV susceptibility (Figure 6). These data implied that NO might be essential component of BR-mediated plant immunity against MCMV infection in maize. 

Long-term co-evolution of plants and pathogens has produced sophisticated mechanisms for defense and counter-defense, which contains the involvement of complex networks of signaling molecules, such as hormones, reactive oxygen species (ROS), and RNS [53,54]. Because of the great differences in pathogen lifestyles and the genetic constitution of the host, it is essential that plants adjust their defense strategies to activate the appropriate defense response by modulating multiple signal transduction pathways [55]. Different signaling molecules-mediated defense pathways can optimize plant responses to activate the most suitable defense strategies against specific invaders [56]. In addition, pathogen infection also has significant effects on host signal transduction pathways. As a counter-defense strategy, many pathogens have evolved methods to tap into these signaling networks to interfere with host defense mechanisms [55]. The balance of hormonal crosstalk strongly influences the outcome of plant-pathogen interactions, including the establishment of effective systemic immunity [57]. Recent investigations have shown that abscisic acid/JA/SA pathway was induced to provide better protection against pathogen infection in grape [58], rice [3], etc. On the other hand, BR signaling pathway was also activated to aggravate pathogen-induced symptoms in *Arabidopsis* [59]. Owing to the ceaseless efforts of many scientists, a more integrated picture is emerging of complex crosstalk and induced hormonal changes that modulate disease and resistance, but some key molecular details of pathogen-host interaction are still unknown [57]. Hence, to explore the functions of hormones and other signaling molecules in plant-pathogen interactions is essential for our understanding of plant immune responses and for designing effective strategies of engineering disease resistance in crops.

## Supplementary Materials

The following are available online at https://www.mdpi.com/1999-4915/11/4/368/s1, Figure S1: RT-qPCR verified the reliability of the transcriptome sequencing results, Figure S2: BRZ inhibited NO accumulation in maize, Figure S3: NO content in the SL1 of MCMV inoculated maize plants after application of Tween-20 (0.02%, mock), c-PTIO (200 μM), and a mixture of c-PTIO (200 μM) and BL (500 μM), Figure S4: NO content in the SL2 of BMV-GFP/ZmNR/ZmDWF4 plants. Table S1: List of primers used in this study, Table S2: Information of RT-qPCR related genes in Figure S1, Table S3: The statistics of MCMV-inoculated maize and mock-inoculated maize libraries. Supplementary File 1: Supplementary explanation of some experimental results. Supplementary File 2: Differentially expressed genes between mock-inoculated and MCMV-inoculated maize plants. Supplementary File 3: GO categories of enriched transcripts in maize leaves in response to MCMV infection determined using GO-seq. Supplementary File 4: KEGG analysis of differential gene expression in MCMV-inoculated maize. RNA-seq data with details of datasets are available on the NCBI Short Read Archive Project—PRJNA527190 (https://www.ncbi.nlm.nih.gov/sra/PRJNA527190).
