# Supplementary material for: Nitric Oxide as a Downstream Signaling Molecule in Brassinosteroid-Mediated Virus Susceptibility to Maize Chlorotic Mottle Virus in Maize
Source: Viruses. 2019 Apr 22;11(4):368. doi: 10.3390/v11040368 (PMC6521138; doi:10.3390/v11040368)
Supplement: Supplementary file 1 [file viruses-11-00368-s001.zip › Supplementary Files/Supplementary File 1 Supplementary explanation of some experimental results 1.docx]

File S1 Supplementary explanation of some experimental results

Table S1 List of primers used in this study.

| Gene name | Primer sequences |
| --- | --- |
| *ZmUBI*-F | GGAAAAACCATAACCCTGGA |
| *ZmUBI*-R | ATATGGAGAGAGGGCACCAG |
| MCMV-*CP*-F | ccatgtccgaaattctgctg |
| MCMV-*CP*-R | gatgcgcacagagttgaacac |
| *ZmACAO*-F | GAATGCTCGCTTGGTGTAGAA |
| *ZmACAO*-R | CTTGGGCTTGCCATAGACAATA |
| *ZmACAO2*-F | CACACATCGAGTCTGTCATCC |
| *ZmACAO2*-R | GATCCTGTCGAGCGCATAAA |
| *ZmADL2*-F | CTGGATGGACAAGGACAAGTAA |
| *ZmADL2*-R | CACAGGGTTTCAGGAACAGATA |
| *ZmBAK1*-F | CCATTGGAAGTCCTCTCTCTTG |
| *ZmBAK1*-R | CTTCAGCTTGTCCTGGATGAT |
| *ZmCDPK*-F | TGGAAGTGGCTGATAATGACAA |
| *ZmCDPK*-R | GGTGCTCCTCACGTTCTATTT |
| *ZmGAPC*-F | CTTCATCAGCACCGACTACAT |
| *ZmGAPC*-R | AACACAGCAACCTCCTTCTC |
| *ZmJAZ4*-F | CAGGCGCTTGTGGACAT |
| *ZmJAZ4*-R | CGCCATCTTGCCCTTCTT |
| *ZmLOX7*-F | CCGACATCATCAAGAGAGACAA |
| *ZmLOX7*-R | CTGAAGGCGCTCGATGTT |
| *ZmNR*-F | GTGCATGATAAACGAGCTGAAC |
| *ZmNR*-R | CGGAGTATGCGTATCCTTTGAT |
| *ZmPR1a*-F | GGCGAGAGCCCCTACTAGAC |
| *ZmPR1a*-R | AAATCGCCTGCATGGTTTTA |
| *ZmPR5*-F | GTCATCGACGGCTACAACCT |
| *ZmPR5*-R | CACGGGCAGAAGGTGACT |
| *ZmRBOHC*-F | TCTTTCTTTCTCCACGCAC |
| *ZmRBOHC*-R | CGCAGCACAATGTTATCGT |
| *ZmRBOHD*-F | GCGGGCAGTACATCTTCGT |
| *ZmRBOHD*-R | GCGGGCAGTACATCTTCGT |
| *ZmRBK1*-F | GAATGGGAGCTAGGTACAAAGG |
| *ZmRBK1*-R | GATGGCAGCCTTCATGTAGATA |
| *ZmDWF4*-F | TGGAGGACAGGCTTGAGAAG |
| *ZmDWF4*-R | GCAAGAGGTCCAGGATCTGT |
| *ZmCPD*-F | GGTCTTCACTGCCGTTCATC |
| *ZmCPD*-R | TTAGCGAGCTCTGATCCTGG |
| *ZmBRI1*-F | GAGCGGCTGTTGGTTTATG |
| *ZmBRI1*-R | AGTTGTGGTGGAGGTATGCC |
| *ZmBAK1*-F | CTGGACAGAGGGCATTTGAT |
| *ZmBAK1*-R | CCAACATCTCCACCTTCTTCTC |
| *ZmBES1*-F | GGTGACGCTGTTGTTTCATTC |
| Gene name | Primer sequences |
| *ZmBES1*-R | GATGCCAAAGATCCAGCTAGT |
| *ZmNR*-silencing-F | CCTCCTAGGTACTTGCCGTACTTGGTTGG |
| *ZmNR*-silencing-R | CTTCCATGGAAGCCGGAGTGCATGATAAA |
| *ZmDWF4*-silencing-F | CCTCCTAGGAGGGAAGGAAGAAGAGGAGC |
| *ZmDWF4*-silencing-R | CTTCCATGGAGGGAGAGAGAAATCCAGAGA |
| XSD198 | CTTGTGTTGCTGAGAAAC |
| XSD199 | TCTTGTAAGAGGTCTGC |


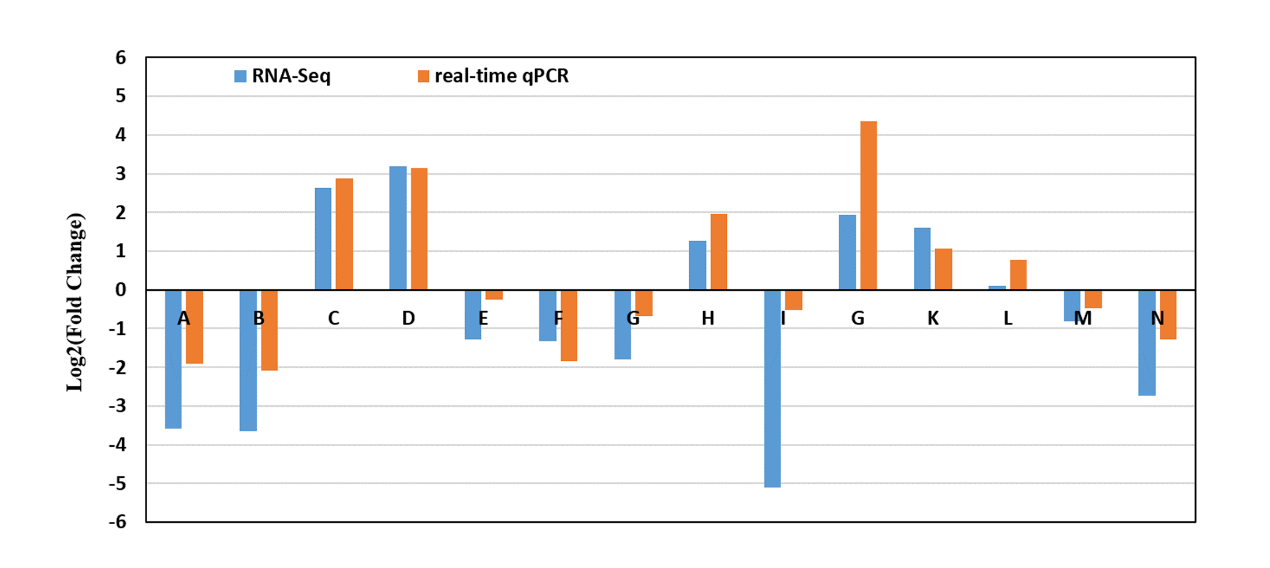


Figure S1. RT-qPCR verified the reliability of the transcriptome sequencing results. Experiments were repeated three times with similar results.

Table S2. Information of RT-qPCR related genes in Figure S1.

| Sign | Family | Gene ID | Anonation |
| --- | --- | --- | --- |
| A | *ZmACAO* | Zm00001d052931 | acyl-coenzyme A oxidase |
| B | *ZmACAO2* | Zm00001d003744 | acyl-coenzyme A oxidase 2 peroxisomal |
| C | *ZmADL2* | Zm00001d027996 | alcohol dehydrogenase-like 2 |
| D | *ZmBAK1* | Zm00001d000298 | BRASSINOSTEROID INSENSITIVE 1-associated receptor kinase 1 |
| E | *ZmCDPK* | Zm00001d027480 | calcium-dependent protein kinase 6 |
| F | *ZmGAPC* | Zm00001d017121 | glyceraldehyde-3-phosphate dehydrogenase |
| G | *ZmJAZ4* | Zm00001d027899 | TIFY transcription factor 1; ZIM motif family protein |
| H | *ZmLOX7* | Zm00001d025524 | lipoxygenase |
| I | *ZmNR* | Zm00001d049995 | nitrate reductase |
| G | *ZmPR-1* | Zm00001d018738 | pathogenesis related protein |
| K | *ZmPR-5* | Zm00001d031158 | pathogenesis related protein5 |
| L | *ZmRBOHC* | Zm00001d038762 | respiratory burst oxidase3 |
| M | *ZmRBOHD* | Zm00001d052653 | respiratory burst oxidase4 |
| N | *ZmRBK1* | Zm00001d018955 | receptor-like cytosolic serine/threonine-protein kinase |

Table S3. The statistics of MCMV-inoculated maize and mock-inoculated maize libraries.

| Sample name | CK^1^ | MCMV^2^ |
| --- | --- | --- |
| raw reads | 64 588 973 | 66 292 567 |
| clean reads | 62 880 365 | 64 567 548 |
| clean bases | 9.43 G | 9.68 G |
| error rate (%) | 0.03 | 0.03 |
| Q20 (%) | 94.84 | 94.24 |
| Q30 (%) | 88.18 | 87.15 |
| GC content (%) | 59.15 | 57.93 |
| total mapped (%) | 84.93 | 83.27 |
| multiple mapped (%) | 4.18 | 3.14 |
| uniquely mapped (%) | 80.75 | 80.14 |
| reads map to '+' (%) | 40.32 | 39.92 |
| reads map to '-' (%) | 40.43 | 40.21 |
| non-splice reads (%) | 53.73 | 52.01 |
| splice reads (%) | 27.01 | 28.13 |

^1^mock-inoculated maize; ^2^MCMV-inoculated maize.


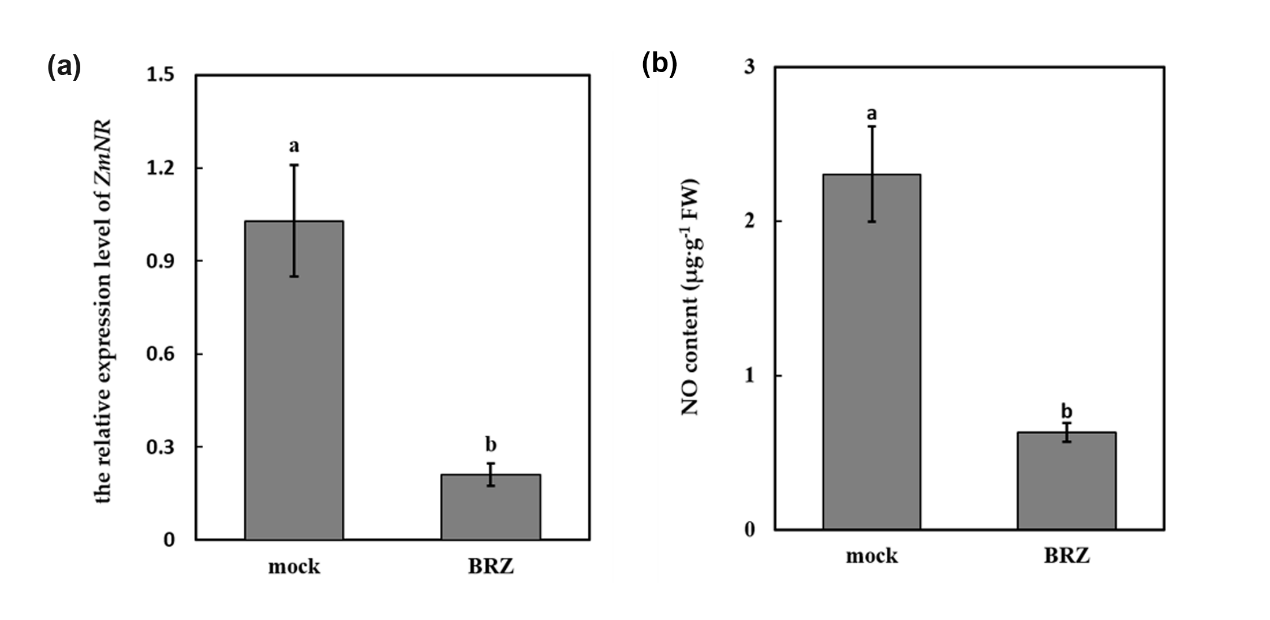


Figure S2. BRZ inhibited NO accumulation in maize. (a) RT-qPCR analysis of the transcript levels of NO synthetic gene *ZmNR*. (b) Quantitative measurements of NO content in the leaves. mock: Tween-20 (0.02%); BRZ: pretreated with BRZ (1 μM). Data are means ± SD from three biological replicates. Statistical analysis was performed using SPSS 16.0 followed by by ANOVA with LSD test at the level of P value ≤ 0.05. Significant difference between different samples was indicated in lowercase letters.


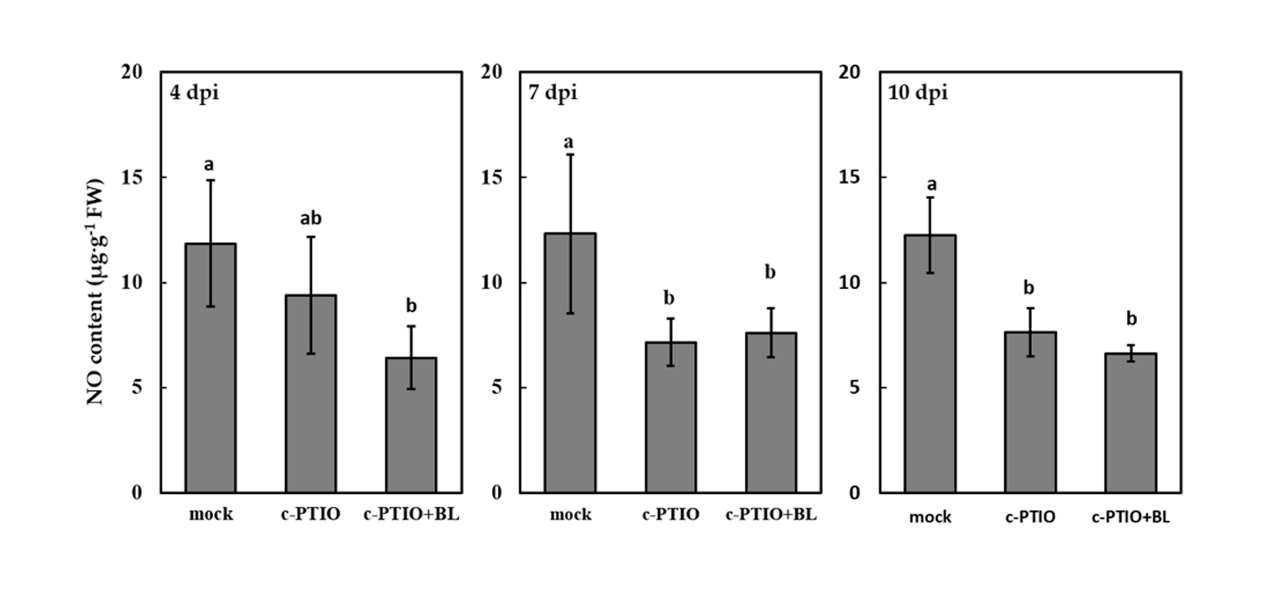


Figure S3. NO content in the SL1 of MCMV inoculated maize plants after application of Tween-20 (0.02%, mock), c-PTIO (200 μM), and a mixture of c-PTIO (200 μM) and BL (500 μM). Data are means ± SD from three biological replicates. Statistical analysis was performed using SPSS 16.0 followed by by ANOVA with LSD test at the level of P value ≤ 0.05. Significant difference between different samples was indicated in lowercase letters.


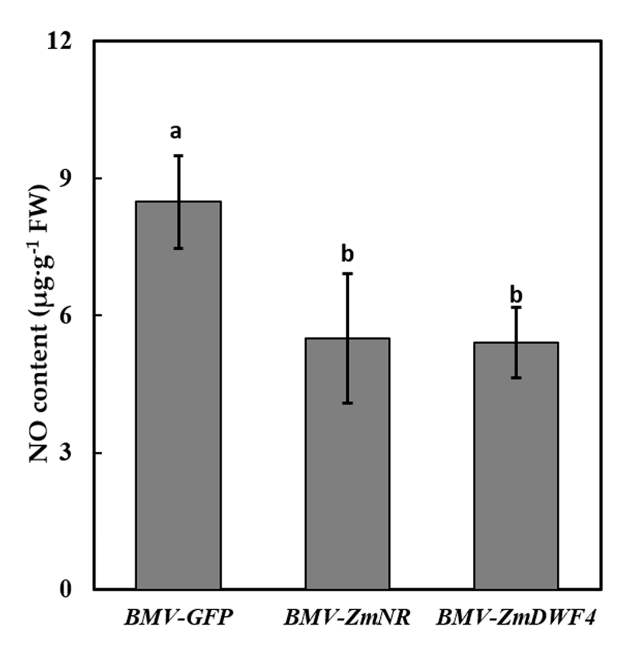


Figure S4. NO content in the SL2 of BMV-*GFP*/*ZmNR*/*ZmDWF4* plants. Data are means ± SD from three biological replicates. Statistical analysis was performed using SPSS 16.0 followed by by ANOVA with LSD test at the level of P value ≤ 0.05. Significant difference between different samples was indicated in lowercase letters.
